# Survey on the Past Decade of Technology in Animal Enrichment: A Scoping Review

**DOI:** 10.3390/ani12141792

**Published:** 2022-07-13

**Authors:** K. Cassie Kresnye, Chia-Fang Chung, Christopher Flynn Martin, Patrick C. Shih

**Affiliations:** Informatics Department, Luddy School of Informatics, Computing, and Engineering, Indiana University, Bloomington, IN 47405, USA; cfchung@iu.edu (C.-F.C.); martincf@indiana.edu (C.F.M.); patshih@indiana.edu (P.C.S.)

**Keywords:** enrichment, animal–computer interaction, animal welfare

## Abstract

**Simple Summary:**

Enrichment is important for supporting the well-being of captive animals. Enrichment increase animal quality of life through encouraging natural behaviours. As enrichment is shifting to a more centered role in animal care, technology is becoming increasingly accessible and is becoming embedded in animal enrichment in creative ways. This review explores the trends in technology usage in animal enrichment studies. Through pulling the past decade of technology enrichment work together, we discuss gaps such as needing to include a larger variety of species (extending passed mammals), ensuring enrichment designs focus primarily on the senses an animal uses to interact with the world rather than human senses, and encouraging similar study designs across animal contexts to allow for streamlined comparisons.

**Abstract:**

Environmental enrichment is adding complexity to an environment that has a positive impact on a captive animal as a necessity of care. Computing technology is being rapidly weaved throughout the space in both enrichment devices as well as evaluating enrichment outcomes. In this article, we present a scoping review of 102 captive animal enrichment studies and propose a contextual lens for exploring current practices. We discuss the importance of directed growth in species inclusion, transitioning beyond anthro-centric designs, and utilizing shared methodologies.

## 1. Introduction

When caring for captive animals, it is important to ensure both physical and mental wellbeing are supported to achieve a high quality of life. Supporting physical health traditionally involves ensuring a balanced diet, access to water, and enough space for an animal to be physically active. Environmental enrichment can aid in supporting an animal’s physical health as well as mental wellbeing through introducing complexity to an environment that is mentally stimulating [1]. Just like humans, animals require mental stimulation to preform a variety species specific behaviors and to encourage exploration and play.

Enrichment “can be broadly thought of as the addition of stimuli or provision of choice that results in the improvement of animal well-being” [2], or simply put adding complexity to an environment that has an overall positive impact on an animal. Enrichment is specific for captive animals, as their captivity simplifies what their environments may have been in the wild, and enrichment aims to bring that complexity back. This includes food, tactical, structural, auditory, olfactory, visual, and social enrichment [3]. These categories are not mutually exclusive, and commonly overlap. Adding complexity back to the environment can fulfil the responsibility we have to the animals in our care, providing them with the balanced environment that surmounts simply surviving [4].

In the 21st century, regular enrichment practices are required throughout captive animal care standards. Enrichment is shifting from a naturalistic approach to one of functional naturalism [5] where stimuli not typically considered in a natural environment are used to stimulate natural behaviors. Enrichment is predominately considered important to animal care, and can be found throughout captive animal lives in laboratories, on farms, in zoos, and in our homes. As new technologies are being released and the information age removed barriers to electronics knowledge, the potential applications for enrichment grow.

A variety of reviews focusing on enrichment have been conducted. A majority of the reviews in the past decade have focused on human-related outcomes with laboratory rodents such as mental health [6,7], physiology [8,9,10], and how enrichment may impact the validity of studies [11]. The growing body of literature also focuses on enrichment with specific groups of animals such as species specific [12,13] or collection of related species such as aquatics [14,15]. Lastly, reviews have also focused on animal care staff perceptions and practices [3,16,17]. These reviews have contributed invaluable insights into the impacts of enrichment for particular species, groups, and medical outcomes. With new technology being developed and accessibility barriers lowering, we want to examine the evolving role technology has in enrichment specifically, both embedded in enrichment devices as well as for measuring objective enrichment outcomes. In this work we aim to take a step back from previous literature focusing on the impact of enrichment and instead broaden the focus see how technology is being incorporated into enrichment studies across animal contexts. These insights may be used to inform future design and evaluation studies, especially for Animal-Computer Interaction (ACI) practitioners.

To examine current trends in enrichment with technology, this review outlines the process used to collect and synthesize the papers, involving a hybrid collection method and affinity diagramming to identify higher trends. Findings regarding the patterns of enrichment in the past decade are discussed, such as commonly used behavioral and physiological methods. We discuss the potential growth of this field, and how practitioners and researchers may need to overcome species favoritism, anthro-centric design biases, and incompatible methodologies. Lastly, this review explores potential research areas for future work to push for more species-centric designs and encourage shared methodologies for generalizability.

## 2. Scope and Criteria for Review

To conduct this review, we utilized snowballing [18] and PRISMA ScR [19]. This involved first selecting a starting set of papers via common search term on different digital libraries followed by iterating between forward and backward citation trees. Each relevant paper was additionally assigned tags to be used for further classifications and aid theme identification. We aimed to capture articles that included animals or animal caretakers to see how studies incorporated a technology component. As technology is temporally defined and inevitably evolves, we focused our definition of technology to be devices that include a computational component such as a touchscreen system or camera recording the enrichment interaction. This allowed us to explore how objective data surrounding enrichment is being collected, whether embedded in the enrichment design directly or if a separate device is used to capture metrics.

### 2.1. Inclusion Criteria

Paper titles were first examined for relevance. Articles containing “animal enrichment” or a specific animal (or animal context, such as farm animals) with welfare terminology (such as animal welfare, wellness, quality of life, etc.) were included. We then considered the abstracts (if not present, the paper automatically moved to the next criterion) and excluded papers that were any of the following:

- Exclusion 1: not relevant to enrichment
- Exclusion 2: were not original and empirical studies (e.g., books, opinion pieces, reviews)
- Exclusion 3: did not include an animal or animal caregiver directly related to enrichment

If an article passed this inclusion requirement, the paper was then fully reviewed to ensure the enrichment focus and inclusion of an animal or animal caregiver directly. The final check was if the paper was published between 2010 and 2020. This time frame was chosen as the beginning of an upsurge for hardware hobbyists with Ardunio in 2008, Raspberry Pi in 2011, and later Phidgits in 2016 all making electronics more accessible to the general public as well as researchers.

### 2.2. Identifying the Starting Set

**Figure 1:**
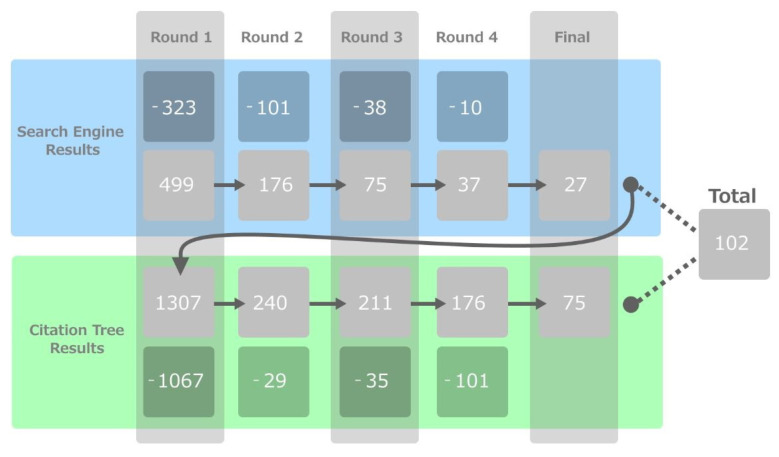
The overall papers included in the review, with points of exclusion for both phases. The blue band represents the search engine round evaluations, and the green band represents the snowballing round evaluations.

We first experimented with the search terms to minimize the presence of unrelated papers in our starting set (including “animal”, “enrichment”, “environmental enrichment”, “behavioural enrichment”, “animal enrichment”). To collect this starting set of papers, the term “animal enrichment” and technology was entered into Google Scholar, ACM Digital Library, and IEEE Xplore Digital Library in March of 2020. All articles returned from that search term were then compared against the inclusion criteria. As seen in Figure 1, this led to 27 papers being collected in the first phase. Forward and backward snowball was then conducted on this starting set, resulting in a total of 102 papers in the final review set (conducted by C.K. independently).

### 2.3. Trend Identification

When reviewing the articles, our main focus was to examine the trends in technology usage in enrichment. Technology was assessed based on if a computational element was present (e.g., camera) and how the technology was utilized (embedded in the enrichment device, or used to collect data to evaluate the enrichment). Tags were used to evaluate the papers through affinity diagramming to identify themes. This was an iterative process, as the tags applied were adjusted while the reading process was conducted. These tags and themes were iterated on with all authors resulting in the discussion points reported in this article.

The works included in this survey are the result of the final set of 102 papers. These papers were reviewed, with tags assigned cyclically to each paper dependent on study design and contributions, as well as article keywords. Those tags were affinity diagrammed and discussed among authors to formulate the trends, hurdles, and future direction.

## 3. Overview of Papers Reviewed

From the papers collected, trends were examined for enrichment over the past decade. A wide array of works were included in this review, with many enrichment types, species, and multidisciplinary evaluation methodologies coming together to shape the area.

### 3.1. Enrichment Technology and Publishing

From the keyword search and snowballing methodology used, a total of 102 peer-reviewed and original studies involving at least one nonhuman animal or animal caretaker published in 2010 or later were collected. This included a total of 52 species, 18 methodologies, and all enrichment types were present. Because enrichment is a cross-disciplinary field, there is the potential for a variety of publication venues. A total of 46 publication venues were included in this review. When looking at the first half of the decade (2010–2014) compared to the second half (2015–2020), the diversity of journals accepting enrichment work increases (16 journals to 20 journals).

### 3.2. Species Present

**Figure 2:**
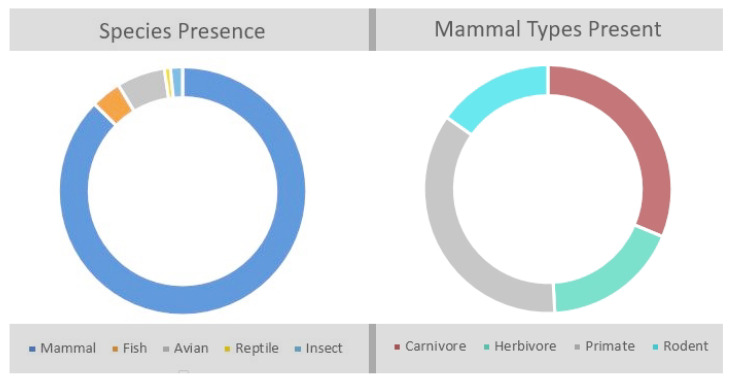
The prevalence of species in the literature.

Along with diverse publishing, a variety of species are present in this work, with 52 total species accounted for. Of these species, mammalian participants are by far the most common class, as shown in Figure 2, with 87% of the participants being mammals. This prominence of mammalian enrichment has been seen in other reviews as well, particularly in zoo contexts [2]. In this group, primates [3,20,21,22,23,24,25,26,27,28,29,30,31,32,33,34,35,36,37,38,39,40,41,42,43,44,45,46,47,48,49,50,51,52] and carnivores [20,21,22,23,25,31,39,40,53,54,55,56,57,58,59,60,61,62,63,64,65,66,67,68,69,70,71,72,72] are the most utilized species. Second to mammals, birds make up a little over 6% of the literature. Much of this work focuses on raptors [25] and forest birds [73,74], with little work in aquatic birds such as cranes [40]. Reptiles [75], fish [76], and invertebrates [77] are all under 3% of the work. No amphibian studies were found, though enrichment studies before 2010 included them [78].

This variety of species is strongly impacted by the study context, as primates and rodents are common in lab studies while ungulates are common in agriculture studies.

### 3.3. Methodologies for Evaluation

A variety of qualitative and quantitative methodologies are present in this body of work. There are four main categories of methods used: behavioral observation, physiological, general metric, and recall-based qualitative inquiry. For behavioral methods overall, the most common method of evaluation is observation, specifically using a pre-crafted ethogram compared to a baseline (such as in the zoo setting, where activity budgets are compared to wild counterparts [63]). Observation-based evaluations were utilized in 61 of the 102 studies (50%). The focal scan-sampling (21 studies, 20.6%) and focal continuous (16 studies, 15.7%) method of observation accompanied with an ethogram are most common, along with some instances of opportunistic, multi-point sampling, and mixed methods [45,66,70]. However, multi-point sampling has been critiqued for the limited ability to capture rare behaviors [68]. Along with ethograms, evaluation tools were also utilized for evaluating enrichment, such as the PAI model [64], tongue-dip ratio [79], and interaction time [45]. Behavioral testing [59,65,77,80,81,82,83,84,85,86] and training [59,67,86] are also commonly used.

Physiological metrics are utilized to varying degrees, but overall are employed throughout the literature. Body condition [87,88,89,90,91,92] and weight [87,90,91,93,94,95,96,97] are common, followed by cortisol (CORT) analysis using a variety of body part such as fecal matter [41,57,69], hair [97], saliva [97], and blood [28,92,97,98,99], as well as surrounding water for aquatic animals [100] and feathers [74]. Some work involved animal sacrifice at the end of the study, resulting in biopsies [81,83,93,96,99] and meat quality analysis [91,101]. Related to physiological metrics, general metrics are also utilized, such as food and water consumption [60,94], distance traveled [76,84,85,87,94], speed [76], pitch of vocalization [82], and game outcomes [29].

For humans included in enrichment studies, most involved in-person interview methods, such as describing the perceptions of enrichment technology for orangutans [37,42] or evaluating their companion animal’s reactions to technology [21,22]. Surveys were also used on targeted groups, such as animal care staff [3,44] and zoo visitors [42], as well as public observations [39]. One instance of focus group methodology was found, exploring a better way to design dog kennels from dog owners, staff, behaviorists, and veterinarians [24].

Related to methodologies, the presence of ethical approval is limited in this area. Of the papers include, over half do not include a statement of ethical approval for human or animal participation.

### 3.4. Computational Technology Usage

**Figure 3:**
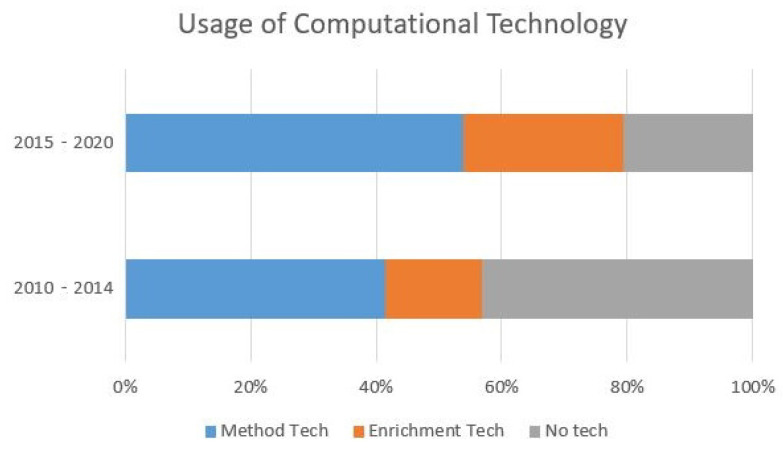
Utilization of technology in enrichment studies between the first and second half of the 2010 decade.

While the definition of technology was relaxed for the filtering stage, a significant portion of the studies included computational technology as shown in Figure 3. This technology can be broken down into two areas: methodology aids and enrichment implementations. Methodology aids are computational technology that is used to aid the facilitation and/or data collection of a study. Examples of this include the use of cameras [30,32,37,45,56,57,71,74,79,81,82,83,88,92,94,102,103,104,105,106,107], observational apps [34,40,51,60,95,108], and tracking software [29,77,93,95,109]. Cameras are the most prominent computational technology to aid in data collection and may present a unique opportunity for computer vision to automate behavioral annotation as manual annotations require significant staff and time resources. On the other side, enrichment implementations have computational technology directly embedded. These include speakers [38,50,52,58,73], feeding devices [31,63,80], and touchscreens [22,23,31,35,42,45,46,54,67]. Touch screens are most common in this area, as the recent improvements in commercial products protect against water and rougher handling of most animals (excluding large mammals such as orangutans, where the technology is usually protected by a barrier).

### 3.5. Animal Contexts

Of the factors differentiating in the present studies, none are more demarcating than the context of the animal. Study contextualization is paramount in informing methodologies, participant choice, and benefiting population in enrichment studies. In this review, five distinct contexts were classified relative to the purpose of the foci animals in the study: Laboratory, Agriculture, Zoo, Companion, and Wildlife. Laboratory studies purchase and breed animals for the sole purpose of experimentation, with almost all aspects of participant life being controlled for study integrity. Agriculture studies have a lesser level of control; however the animal’s purpose is to produce a product (such as meat or milk) which drives the measurements used. Zoo studies provide the widest range of species and balance goals of human education, conservation, and animal welfare with public perceptions as the animals are traditionally on display for large gatherings. Companion animal studies explore a snapshot into the relationship between an animal and their human counterpart, where the animal’s purpose is related to the human companion’s well-being. Lastly, wildlife studies are the most uncommon as enrichment is a captive activity and only rehabilitation places wildlife in a captive state temporarily. An important distinction for wildlife rehabilitation compared to the care provided in other contexts is the aim of reintroduction to the wild without human familiarization [36]. Applying a contextual lens to enrichment technology literature allows us to explore the nuances and potential hurdles while remaining aware of the animal experience and human motivation of each context.

## 4. Contextual Overview

The contextual distinction may be drawn through research questions alone, with added clarity given by species and methodologies choices. In this section, the distinct factors and findings of each individual context are outlined, with commonalities highlighted.

**Figure 4:**
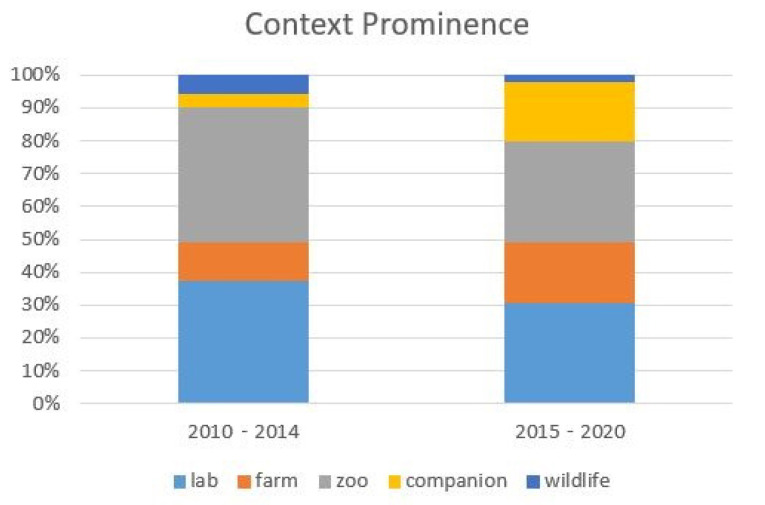
The distribution of contexts present in the literature as divided by the first and second half of the decade.

The distribution of these five contexts in the literature can be seen in Figure 4. The laboratory and zoo-dominated field has equalized as the decade progressed, with more companion and farm animal studies being conducted. The wildlife setting decreases tremendously, with only one study occurring in the second half of the decade.

### 4.1. Laboratory

The laboratory setting of enrichment work aims to predominately aid human health outcomes [83,86,87,93,95,98,110,111,112,113], expand general behavioral knowledge [29,59,77,80,81,84,85,96,99,109,114,115], and improve animal welfare [30,31,32,33,35,60,74,82,94]. Laboratory work tends to be dominated by rodent participant usage, as shown in Table 1. Along with rodents, primates, birds, fish, and insects were also used as participants in laboratory studies in varying amounts.

The enrichment being evaluated in the laboratory focuses on object [28,32,34,60,74,77,80,81,83,84,85,86,93,94,95,98,99,109,110,111,113,115] and structural [32,34,77,81,83,84,85,86,87,93,94,95,96,98,99,109,110,111,113,115] methods, usually characterized by enriching an environment through additional ramps and shelters with plastic pet store toys, along with notable inclusion of touchscreen [29,31] utilization. Social enrichment [28,80,83,84,85,94,99,109,110] is also used in a lesser capacity with a focus on social intra-species behaviors, though work has also been explored using social decoys [76] and animal–human interaction [82]. Food is moderately used as enrichment [29,31,32,33,59,60,77], though is usually paired with training [30,33,59] and is never used alone. Videos [35] and spatial [114,115] enrichment are scarce, with only one study of either being found. No focal olfactory or auditory studies were found in this context, though effects of these stimuli may have impacted social findings. When looking at social and object enrichment, a mix of both was found to have the greatest positive effect on immunity [28] as well as better cognitive performance [80].

For methodologies, the laboratory setting utilizes behavioral testing [29,31,35,59,83,84,85,86,94,109,110,111] and observation often. Observation methods utilized include focal animal [30,32,33,34,95], instantaneous scan sampling [32,35], continuous [33,60,95], all occurrence [32], opportunistic [77], and unspecified [82,94,98,115]. Related to observation, vocalization range [82], social spacing [76], and movement patterns [76,77] have also been used to determine the impacts of enrichment. Physiological metrics are also important, as biopsies [83,95,96,114] are used (usually looking at brain structure [81,83]), as well as distance traveled [84,85,94], food and water consumption [60,94], weight [94], blood [98], and body condition [98]. Studies focusing on animal choice are rare [60], and interview methods for stakeholders was only utilized by one study [31].

Most technology deployed in the laboratory context is through operant chambers (enclosures with built in interactive mechanics for behavioral testing) [76,77,80,84,85,86,94,111], and cameras for recording behaviors [30,32,74,76,81,82,83,87]. Touchscreens [29,31] and feeders [31,80] are used less often. Lastly, microphones [82] and observation application [60] utilization are limited to a single occurrence in this context. Of these studies, most of the technology used was commercial, with some exceptions [29,31,76,77].

When looking at the outcomes of the laboratory studies, there is predominate evidence that across the board enrichment has a significant impact on animal behavior and physiology [28,29,31,32,35,60,77,80,82,83,84,85,86,87,94,95,98,99,109,110,111,112,113,114,115], with some instance of little to no impact [30,33,34,35,59,94,96]. Enrichment increases the impulsivity [80,85,110] and behavioral variability [77] of animals, and overall improve welfare [32,82,87,113,115]. A few studies found specific significant results when looking at results at the individual scope, and others found a strong impact of their proposed system [29,31,76].

The laboratory context separates itself from other contexts through the common research aims of human health and cognitive behavioral understanding while still holding welfare high, as seen with studies suggesting improved methods of including animals in studies [116,117]. The participants in these studies tend to be rodents and primates, as shown in Table 1, though a variety of species are included. Enrichment types tend to focus on object, structure, and social, with a couple of exceptions of training as enrichment to lower stereotypic behaviors. Evaluating these enrichment methods tend to involve behavioral tasks and direct observation. The technology used in this context is commercial and is commonly video and audio recording that requires automatic or manual coding.

### 4.2. Agriculture

Animals in agriculture are included in enrichment studies with common goals of improving product quality and minimizing injury cause by farming practices or intra-species aggression. A good portion of this work is motivated by changes in legislation [26,89,92,118]. Along with this, motivations to improve animal welfare are common [90,91,97,105,106,119] and care efficiency [27,102,104,107,119] with some focusing on financial benefits [88]. Studies included in this context can be found in Table 2.

To examine this area, the agriculture context uses observation most often. While details are not always given for the kind of observation method used [88,91,97,105,106,107,118], the use of scanned sampling [89,90,102,104,119] and continuous sampling [92] were found. Following observation, body measures are used. Body weight [89,90,91,92,97,105] and condition [88,90,91,97] are common, with the use of CORT levels from hair [97], blood [92,97], saliva [97], and fecal matter [119]. Meat quality is also examined [91]. For human participants, survey methods are used [26,27].

The enrichment examined in this area is predominately exploring the use of straw, which serves as both object and food enrichment [88,89,90,91,92,102,104,105,107,118]. In addition to exploring its effect, Wallgren et al. surveyed farm owners and found that 98% of farmers use straw, with only 37% supplying another enrichment object [27]. This same survey also found that the common concern of straw being difficult to work with was not the case, as the farmers reported few instances of straw obstructing the pens, which supported using enrichment over tail docking in pigs (as tail biting is a common aggressive behavior and is lessened when enrichment is used). Along with this, Statham et al. found the timing of distributing the straw enrichment was not important [90], and Machado et al. noted removing objects overnight or alternative days of enrichment was not effective at keeping animal interest [102].

Structural enrichment such as slated floors is also reviewed in this area [92,102,118,119]. There are also two instances of olfactory enrichment examined, one that was paired with straw [102] and one that was a vanilla scent alone [105]. Overall, enrichment was found to result in lower animal stress as seen through CORT samples and reduced aggressive behaviors [88,89,97]. Effects of reduced CORT levels and aggression were not always paired, as Cornale et al. found both lessening animal density and object enrichment were needed to lower both levels [119]. The effects of enrichment are short term, as regular enrichment is necessary to ensure lower stress in animals [106].

### 4.3. Zoo

Zoos (seen in Table 3) have shifted over the past century from a place of entertainment to education and conservation. These changes ushered in new enclosure designs and a stronger focus on animal welfare [41]. Many of the studies in this context are motivated by improving welfare [37,38,39,41,64,65,108,120], with a few including zookeeper considerations [37,63].

This focus on welfare has produced a variety of enrichment strategies explored in this context. Food [40,63,64,65,79,120] and object [40,64,65,66,79,120] are the most utilized (as also noted by [3]), with auditory [38,52,73] following. Structural [40,65,103], visual displays [37], and social interactions [65,103] (including animal–human interaction [39,43,108]) are less common.

These enrichment types are evaluated almost entirely through observation. Scan sampling is commonly employed [38,43,65,108], as well as continuous focal sampling [43,51,52,72,103]. Some did not specify the observation type [40,63,79], and one used a behavioral scoring system [66]. Some other methods used in this area are interviews [41] and surveys [3].

The computational technology used in this area focuses on aiding the observation process by using cameras [37,79,103] and speakers [38,52,73] as well as mobile ethogram apps [40,51,108]. A few custom systems were created such as an orangutan projected game [37], an automated feeder [63], and a chimpanzee music selector [38].

### 4.4. Companions

Companion animal enrichment (shown in Table 4) has a large potential for financial growth, with a new awareness being given to pet care. Studies in this area of motivated by a species-specific approach [22,23,24,56,58], fostering animal–human interaction [22,23,25,54], welfare [21,25,57], and standardizing evaluation methods [55]. Enrichment examined in this area focuses on visual displays [22,23,54,56], with auditory cues [21,58] and objects [21,55] moderately used, and solely animal–human interaction [25] also explored. Methods of evaluation tend to be general observations [21,22,23,54,56,57,58], with human-focused methods such as interviews [21,25], surveys [25], and focus groups [24] also used. Technology utilized is usually for visual purposes like tablets [22,23,54] and projectors [56] or cameras [56,57], activity trackers [25], and speakers [58]. While the tablet and projected designs usually encompass custom software, the only custom hardware project found in this area is a button [55] for canine interaction. The general findings in this area support work for facilitating animal–human interaction and provide insights for future designs [22,23,24,25,54]. There was a significant impact on evaluated behaviors found [21,57,58], and one review of an evaluation framework with positive results [55].

### 4.5. Wildlife

Wildlife is the least represented group in the literature (seen in Table 5). As discussed earlier, enrichment is for captive animals unable to live out wild lives, and wildlife is rarely in a captive capacity. The exclusion of this is for rehabilitation purposes, as well as sanctuaries. Rehabilitation targets a reintroduction to the wild, meaning care must be monitored to ensure a successful transition. However, some wild animals cannot be released due to injury or familiarization, which has them transition to sanctuary animals. These animals are different than zoo animals, as most aspects of their care remain the same as rehabilitation, without the minimizing human contact emphasis, but limiting public contact with the animal. In this area, two studies from each of the subgroups were found. For rehabilitation, motivations are of encouraging natural behaviors [62] and examining technology needs [36], and technology has been proposed to this end including a smart habitat system [36]. Sanctuary animal studies are motivated by welfare [61,75] and understanding wild behaviors [61], with no technology currently being used. Enrichment types in this area are expansive, including temperature [36], structural [36], food [61,62], olfactory [61], object [62,75], and auditory [62]. Most interesting was the use of animal–human interaction [62,75], though was only for interactions with care staff for animals in sanctuaries. Evaluations all use general observations [36,61,62,75], with some participatory observation [64] and choice testing [61] utilized. Overall, enrichment is found to aid animal welfare [61,62,75], and there is a need for more technological support in care and management [36]. These studies tend to have smaller sample sizes due to the collection process, and individual differences are still seen [62,75].

## 5. Trends

The process of evaluating the past decade of enrichment studies related to technology provides a glimpse into the potential of the area as a whole. Enrichment studies are propagating into new publication venues, with broader inclusions of species, and increased cultivation of technological devices and measurements. The positive growth seen across animal contexts shines as an augury for the future of enrichment. This area has been germinating for over 50 years, and recent sprouting caused by increased accessibility and affordability of technology will need to address impending challenges of species diversity, anthro-centric designs, and contextual divides limiting flow of information and animal welfare.

### 5.1. A Blossoming Field

The literature review presented here overall shows a promising future for technology in enrichment research. The wide variety of species explored in these studies highlights the breadth of enrichment application across contexts and encourages even more inclusion for future work. For ACI practitioners in particular, the use of computational technology has overall increased. This increase in computational technology utilization hints at the potential demand for more enrichment technologies that nontechnical users can administer.

#### 5.1.1. Steady Stream of Studies and Increased Venues

When looking at publication venues of the papers in this review, there is not only a growth in the total number of studies being performed, but the range of venues where the work is being submitted and accepted, as has been also seen by earlier literature reviews [17]. While the number of studies sent to each venue fluctuates, the increased diversity of journals accepting enrichment studies may show an increased quality of the studies as well as an improved awareness of the necessity and merit of enrichment.

This increase in journal diversity and studies is seen across and within contexts. This indication signifies the importance of research within each context, with most areas being increasingly published. This increase in publication may be correlated with the inclusion of computational technology allowing for more enrichment device designs as well as study methodology aids.

#### 5.1.2. Increased Technology Being Utilized

While computational technology is gradually becoming more common in enrichment devices, measurement technology for methodology is growing rapidly. As shown in Figure 3, the percentage of studies that contain a computational technological component is increasing, with the largest increase for methodology enhancing devices. This overall shift in methodology and materials may be further supported with ACI devices and evaluation frameworks.

Computational technology embedded in enrichment systems are still in early stages. Contextually dependent increases do exists, such as the use of touch panels in zoos or projections for companion animals, however this number has experienced a more gradual increase. In many cases, the financial burden of incorporating technology can be high, as it includes the cost of the technology, the cost of hiring an expert, and the cost of upkeep [122]. These financial concerns are weighed against the potential positive benefit for the animal, which may be limited by novelty effects. This cost-benefit analysis may be why touch panel technology has had the most drastic increase, as touch panel interactions can be adjusted for novelty through different games presented on a single hardware setup as well as a wide variety of species are able to interact with the same base device.

#### 5.1.3. Increased Species Inclusion

While differences in the frequency of species can be seen contextually, the overall growing diversity across the field is promising. This increase of species inclusively allows for interdisciplinary work combining technology and animal experts, and consequently opens the door to more publishing venues and funding. By working with a variety of experts, more domain specific publication venues that rely on implicit knowledge are reachable through the improved awareness as both reviewers and authors.

This increase is not equally distributed across contexts, with a strong focus remaining on mammalian species as was also found in 2015 by Alligood et al. [17]. While species diversification can be difficult in specific contexts, species diversity will need to be addressed in the contexts that are able to include nonmammalian species.

### 5.2. Shared Challenges

The sprouting and growth of this field is not without challenge. As this area gains momentum and explores new avenues, the growth needs to be directed to include a larger variety of species, move away from anthro-centric designs, and utilize shared methodologies. Encouraging a strong foundation across contexts for this growth will allow the field to reach new heights and aid animal welfare.

#### 5.2.1. Species Diversity

The species present in the literature is growing, but is not all inclusive as the literature favors mammalian participants. This exclusion in part can be connected to limited contextual access, the difficulties of exploring enrichment in a species’ environment (e.g., Ocean, Atmosphere), or the perceived sensory limitations of species.

The first limitation of access is directly connected to government regulations that cannot and should not be loosened. Recommending that these contexts specifically incorporate more species in their studies does not consider context specific aims. While some contexts have a larger pool of species to work from, others have strict regulations that legally, practically, and ethically bar the inclusion of different species (such as care guides [117,123]).

The second limitation is environmental hurdles, which is commonly seen in a zoo setting. The financial and temporal requirements of crafting and evaluating enrichment can be resource heavy in grassy enclosure, and becomes even more complex when applied to a water enclosure.

Perceived animal consciousness is an implicit hurdle, as enrichment is usually given to species that are easy to anthropomorphize like mammals. Enrichment can and should be tailored to cognitive ability, however different cognitive capacities should not exclude a species entirely from enrichment research. Perceived consciousness is the most important hurdle to overcome regarding species inclusively. Through exploring all aspects of the animal kingdom, more technological challenges can be uncovered and conquered, more interdisciplinary connections can be made, and steps can be taken towards high quality of life equally.

#### 5.2.2. Move Away from Anthro-Centric Design

As discussed previously, some species have government and organizational requirements imposed that limit procedures possible. However, an implicit limitation is also seen across contexts to enrichment perceivable by human senses as well. This anthro-centric limitation places a bigger focus on visual and auditory changes to an environment, and rarely explores enrichment through a species-centric enrichment selection process. Notable exceptions to this observation can be found such as the MEAU method [55] and calls for more inclusive enrichment activities [124]. This can be seen using visual enrichment in particular, as many studies do not take into account the refresh rate of the visual displays.

When a species-centric approach is taken, the stimuli pool tends to be limited to senses shared by both animals and humans. This may be a consequence of evaluation metrics that are design for a human to review. However, this vastly limits the ways we enrich animals and may keep hidden unknown healthy behaviors that are a direct response of a stimulus unperceived by the human body. Instead of limiting stimuli to be only shared senses, researchers can examine new technology to aid in evaluation methods and allow an animal to fully connect with their environment with all their senses.

#### 5.2.3. Incompatible Methodologies

The last shared hurdles for enrichment research is the differences in methodologies employed across contexts. Section 4 describes the nuances of methodology choice contextually, resulting in a difficulty to compare results across contexts. Similar methodologies are a necessity for generalizability, and need to be shared between contexts that study similar species. While aims of a study may be for future human application, or to further improve a rearing process, that information may still be important to the species well-being that can be disseminated through contexts.

Transferring work between contexts where a species is present is vital to animal welfare [16], and incompatible methodologies stands as a road blocker to transmission. Turschwell et al. recreated enrichment strategies commonly used with mosquito fish and found the wild caught population had drastically different physiology and behavior than the laboratory standard supply [114]. This shows the importance of evaluating species in similar manners across contexts, as welfare concerns and behavioral findings will become easier to spot.

## 6. Future Direction

The past decade of enrichment and technology work has shown the effectiveness and importance of enrichment in animal quality of life. Although challenges such as species diversity, anthro-centric design, and methodology incompatibilities need to be addressed; the future of this field remains bright. To ensure a positive course of action, researchers and practitioners may utilize species-centric designs that are evaluated with shared methodologies and disseminate this information in a common format.

### 6.1. Taking Species-Centric Design a Step Further

The anthro-centric design challenge to this area can be circumvented by taking extra care to design for non-human senses specifically. Using a non-human sense will encourage technological innovation to be able to evaluate the effectiveness and impact of the new enrichment veins. By placing the focus on senses that are not shared, this may also aid the designer in taking a new perspective for the species specifically and minimize human sense biases. Taking an individualized approach will also be a necessity [55,125,126]. Enrichment, much like quality of life, is only able to be evaluated through the perception of the subject. While generalized enrichment such as puzzle feeders can be deployed for an entire species, the devices and systems can be enhanced to fit the specific target user on an individualized level. This level of specialization will require more work in preference for animals, and methods for determining preference. Once preferences are understood, designs can be maximized to not only focus on species-centric senses, but include individualized enhancements such as favorite food during the enrichment activity as well.

Lastly, special care should be taken when designing enrichment to focus solely on the targeted sense. For example, while an olfactory object may be targeting to stimulate smell, the color or sounds of the container may have confounding impacts on the animal. By approaching the design through a species-centric approach, researchers can understand the range as well as limitations of a species sense. Using this knowledge, designs can be crafted to emphasize the target stimulating factor, while minimizing accidental stimuli. An example of this can be creating a ball that has a good mouth feel for a teething puppy, while using colors that is less stimulating. Similar hybrid design ideas may be applied in the zoo setting by focusing enrichment on stimulating targeted sense, while using minimal or undetectable stimuli to appeal to visitors.

### 6.2. Methodology Network

There is a need for an established set of tools that a wide variety of researchers can use, to allow for intercommunication of impacts as well as strengthen the reliability of the studies. While observation was found in all contexts, the focus of the observation differed between contexts. This isolates findings to only be comparable to animals within the context and may not hold true for the same species in different contexts [114].

One way to standardize evaluations in studies is through the creation of computer vision models for behavioral annotation that can be shared [127]. Most of the studies included in this review utilize some kind of video data. This data is then coded by hand based on an ethogram the study is using. Instead, the resource heavy annotation phase can be replaced with a computer vision model that can be run on data sets from a variety of contexts. This may support volunteer heavy contexts such as wildlife rehabilitation and animal shelters that have limited resources.

Another standardization can be through embedding enrichment devices with data collection capacities. The works presented in this review relied on data collection such as cameras and timers that could be included in future iterations of the enrichment device itself. This would also open opportunities for cross context and species comparisons through deploying the same enrichment device.

### 6.3. Limitations

This review is limited by the search terms used to collect the initial paper set and the accessibility of papers behind paywalls.

## 7. Conclusions

Through a hybrid review, we explored trends of technology in enrichment studies. The field as a whole appears to be blossoming across animal contexts such as laboratories, farms, zoos, companions, as well as wildlife. While challenges remain of species diversity, anthro-centric design, and incompatible methodologies, the field shows promise in utilizing computational technology to both aid in study methodologies and embed in enrichment implementations. The future of this field may benefit from pushing species-centric design further and standardizing data collection such as including computer vision annotations to further generalizability.

## Figures and Tables

**Table 1:** Overview of the laboratory setting. The mean study length column represents the average time (in days) of study procedures. Shading added to aid readability.

Species Present	Studies	Method Category	Mean Sample Size	Mean Length of Study
Chimpanzee	[29]	Behavioral [29], General [29]	2	-
Dog	[31]	Behavioral [31],	265	-
Fruit Fly	[77,112]	Behavioral [77], Physiological [112], General [77]	-	25
Grizzly Bear	[59,60]	Behavioral [59], General [60]	7	142
Guyanese squirrel monkeys	[32]	Behavioral [32]	7	70
Human	[31]	Inquiry [31]	-	-
Macaque	[28,30,33,34,35]	Physiological [28,33], Behavioral [33,34,35]	46	73
Mice	[87,93,95,109,113,115]	Physiological [87,93,95], Behavioral [93,95,109,115], General Metrics [87,115]	58	132
Mosquito Fish	[114]	Physiological [114]	291	-
Nutcracker	[74]	Behavioral [74], Physiological [76]	41	223
Rat	[80,81,82,83,84,85,86,94,99,110,111]	Behavioral [80,82,83,84,85,86,94,99,110,111], Physiological [81,83,94,99], General [82,84,85,94]	46	48
Pig	[98]	Behavioral [98], Physiological [98]	28	55
Stickleback	[96]	Physiological [96]	-	182
Wolf	[31]	Behavioral [31]	20	-
Zebra fish	[76]	Behavioral [76], General [76]	40	180

**Table 2:** Species and measurements break down for farm animals. The mean study length column represents the average time (in days) of study procedures. Shading added to aid readability.

Species Present	Studies	Method Category	Mean Sample Size	Mean Length of Study
Human	[26,27]	Inquiry [26,27]	123	-
Lamb	[101]	Physiological [101]	60	35
Pig	[88,89,90,91,92,97,102,104,105,106,107,118,119]	Physiological [88,89,90,92,97,102,106,119], Behavioral [88,89,90,91,92,102,104,106,107,118]	332	57

**Table 3:** Overview of zoo animal inclusion. The mean study length column represents the average time (in days) of study procedures. Shading added to aid readability.

Species Present	Studies	Method Category	Mean Sample Size	Mean Length of Study
African Wild Dog	[69]	Behavioral [69], Physiological [69]	2	75
Bobcat	[65]	Behavioral [65]	4	180
Brown Bear	[64]	Behavioral [73]	10	60
Capuchin	[121]	Behavioral [121], physiological [121]	10	4
Cheetah	[68]	Behavioral [68]	8	16
Chimpanzee	[38,45]	Behavioral [38,45]	15	206
Condor	[120]	Behavioral [120]	1	203
Coyote	[40]	Behavioral [40]	-	-
Crane	[40]	Behavioral [40]	-	-
Eagle	[120]	Behavioral [120]	4	203
Fox	[40,63]	Behavioral [40,73]	10	60
Gibbons	[52]	Behavioral [51]	8	42
Giraffe	[79]	Behavioral [79], General Metric [79]	8	90
Goat	[108]	Behavioral [108]	15	180
Gorilla	[45,46,50]	Behavioral [45,46,50], General Metric [46]	2	65
Human	[3,39,40,41,42,43,44,48,49]	Behavioral [39,43], Inquiry [3,39,40,41,42,44,48,49]	162	-
Jaguar	[39]	Behavioral [39]	-	-
Llama	[108]	Behavioral [108]	16	180
Leopard	[39]	Behavioral [39]	-	-
Lion	[39]	Behavioral [39]	-	-
Macaque	[45,47]	Behavioral [45,47]	4	95
Maned wolf	[71]	Behavioral [71], General Metrics [71]	8	0
Orangutan	[37,42]	Behavioral [42]	5	118
Otter	[40]	Behavioral [40]	-	-
Parrot	[40,73]	Behavioral [40,64]	3	-
Penguin	[43]	Behavioral [43]	25	10
Pig	[108]	Behavioral [108]	6	180
Polar Bear	[66]	Behavioral [66]	1	119
Puma	[39]	Behavioral [39]	-	-
Ray	[103]	Behavioral [103]	14	240
Sloth Bear	[70]	Behavioral [70]	14	75
Spectacled Bear	[72]	Behavioral [72]	2	365
Sun Bear	[53,67]	Behavioral [53,67]	3	169
Tamarin	[51]	Behavioral [51]	4	180
Tiger	[39]	Behavioral [39]	-	-
Vulture	[120]	Behavioral [120]	4	203
Zebra Fish	[100]	Behavioral [100], Physiological [100]	-	7

**Table 4:** Species and measurements break down for companion animals. The mean study length column represents the average time (in days) of study procedures. Shading added to aid readability.

Species Present	Studies	Method Category	Mean Sample Size	Mean Length of Study
Cat	[22,23,56,57,58]	Behavioral [22,23,56,57,58], Physiological [58]	16	11
Dog	[20,21,25,54,55]	Behavioral [21,54,55]	14	7
Human	[20,21,22,23,24,25]	Inquiry [20,21,22,23,24,25], General [25]	11	21

**Table 5:** Species and measurements break down for wildlife animals. The mean study length column represents the average time (in days) of study procedures. Shading added to aid readability.

Species Present	Studies	Method Category	Mean Sample Size	Mean Length of Study
Coyote	[61]	Behavioral [61], General Metric [61]	16	-
Human	[36]	Inquiry [36]	13	180
Maned Wolf	[62]	Behavioral [62]	3	365
Tortoise	[75]	Behavioral [75]	3	60

## Data Availability

Not applicable.

## Abbreviations

The following abbreviations are used in this manuscript:

ACI	Animal–Computer Interaction
